# Patient preferences in papillary thyroid microcarcinoma management are driven by aversion toward complications rather than treatment pathway

**DOI:** 10.1016/j.surg.2025.109694

**Published:** 2025-09-25

**Authors:** Rebecca Kowalski, Kendyl Carlisle, Aprill N. Park, Salome Ricci, Reuben Don, Carrie Cunningham, Julia F. Slejko, C. Daniel Mullins, Yinin Hu

**Affiliations:** a Department of Surgery, University of Maryland School of Medicine, Baltimore, MD; b University of Maryland School of Medicine, Baltimore, MD; c Department of Practice, Sciences, and Health Outcomes Research, University of Maryland School of Pharmacy, Baltimore, MD; d Department of Practice, Sciences, and Health Outcomes Research, University of Maryland School of Pharmacy, Pharmaceutical Research Computing (PRC), Baltimore, MD; e Department of Surgery, Mass General Brigham, Boston, MA

## Abstract

**Background:**

Papillary thyroid microcarcinomas carry an excellent prognosis, making patient preferences and cost-effectiveness important determinants of treatment selection. To conduct cost-effectiveness analyses, quality-adjusted life year weights for papillary thyroid microcarcinoma treatments must be derived. Our objective was to estimate the quality-adjusted life year weights of common papillary thyroid microcarcinoma treatment scenarios.

**Methods:**

This study used 10 previously published papillary thyroid microcarcinoma clinical vignettes describing active surveillance, radiofrequency ablation, partial thyroidectomy, and total thyroidectomy, along with potential complications (progression, vocal cord palsy, hypocalcemia). Quality-adjusted life year weights were derived using a time trade-off instrument administered to thyroid cancer survivors. Quality-adjusted life year weights were compared using within-subjects repeated measures analysis of variance and paired Wilcoxon rank-sum tests. The cohort was powered to detect a minimal important difference with an effect size of 0.5 (ie, 0.04 quality-adjusted life year).

**Results:**

Data from 101 thyroid cancer survivors were collected. Median quality-adjusted life year weights for uncomplicated treatment scenarios ranged from 0.975 to 0.992 and were not significantly different between treatments (*P* = .15). Treatment complications resulted in significantly lower quality-adjusted life year weights across all treatment strategies (*P* < .01) except active surveillance (*P* = .72).

**Conclusion:**

Quality-adjusted life year weights were comparable between the uncomplicated versions all 4 treatment pathways, suggesting that patient treatment preferences for papillary thyroid microcarcinoma are driven by aversion to treatment complications, rather than an inclination toward the experience of the treatments themselves. These quality-adjusted life year weights may be readily incorporated into value assessments for papillary thyroid microcarcinoma treatments.

## Introduction

Papillary thyroid microcarcinomas (PTMCs) are small (<1 cm), low-risk thyroid cancers that carry an excellent prognosis.^1^ The treatment paradigm for these cancers is evolving as evidence mounts that less aggressive strategies result in similar survival to more traditional approaches. Active surveillance, in which cancers are monitored by ultrasonography, has shown promise as an alternative to resection.^2–4^ Although not yet standard of care for PTMC in the United States, ablative techniques have demonstrated promising results in other countries.^5,6^ Given this shifting landscape, it is important to evaluate the cost and resultant quality of life of these treatments that are unlikely to yield meaningful differences in survival.

To conduct cost-effectiveness analyses (CEAs), patient preferences must be incorporated to reflect the value of each treatment. This is most often achieved by using quality-adjusted life years (QALYs), which adjust survival time by the quality of life associated with the health state during that survival time.^7^ To date, CEAs for low-risk thyroid cancer have been limited by the QALYs they have incorporated. Some have used QALYs derived from generic quality of life surveys (eg, 36-Item Short Form Health Survey or Euro-Qol–5 Dimensions), which have been shown to have limited responsiveness and significant ceiling effect in thyroid cancer.^8–14^ Other CEAs have incorporated QALYs that were estimated with gold standard techniques (eg time trade-off surveys, standard gamble) but used general population participant samples.^15,16^ Additionally, QALY estimates have yet to be derived for the emerging treatment approach of percutaneous ablation.

The objective of this study is to estimate the QALY weights of common PTMC treatment scenarios that can be incorporated into cost- and comparative-effectiveness research. We hypothesized that, because most PTMC treatments are well-tolerated, QALY weights would be comparable across treatment strategies in the absence of major complications.

## Methods

### Study design

We conducted a cross-sectional study in which participants completed a time trade-off (TTO) instrument and an optional demographics survey. The TTO instrument consisted of preference assessments for 10 PTMC health states, including 4 uncomplicated treatment scenarios (active surveillance, RFA, partial thyroidectomy, total thyroidectomy) and 6 complicated treatment scenarios (temporary and permanent nerve injury, permanent hypocalcemia, cancer progression). The 10 health state descriptions were collaboratively created by a community advisory board composed of thyroid cancer survivors, cancer survivor caregivers, and physicians. Their development and the full health state descriptions are detailed in a prior study.^17^ Health state vignettes include the diagnosis, the treatment performed, symptom profile before and after treatment, complications, medications and surveillance, scar characteristics (if applicable), risk of cancer recurrence, and quality of life factors.^17^

Individual participants completed study instruments during a standardized video conference session proctored by a trained research assistant. For each of the 10 thyroid cancer health states, participants were presented with one vignette describing the health state and another describing perfect health. Then, they were asked to indicate how many months or years (if any) of the 10-year survival time in the described cancer health state they would be willing to give up to instead live with perfect health. In this way, a QALY weight is derived for each PTMC vignette, where 0 = death and 1 = perfect health. For example, the QALY weight for an imperfect health state in which the participant indicated they would consider 10 years in that health state equivalent to 9 years with perfect health would be 0.9.

### Participant selection

Patients previously treated for early-stage, differentiated thyroid cancer were recruited from (1) our institution and (2) ResearchMatch.org, which is a national web-based recruitment tool created through the NIH Clinical & Translational Science Awards Consortium as an institutional review board–approved data repository.^18^ For the prior, we retrospectively searched our institutional tumor registry and electronic medical records for patients diagnosed with early-stage thyroid cancer between January 1, 2015, and December 31, 2022. Those with personal history of thyroid cancer, fluent in English, and 18 years of age or older were invited to participate. Individuals were excluded if they lacked the mental capacity to answer study questions. There was no inclusion or exclusion criteria regarding sex of participants. Because of limited PTMC patient volume, the study cohort was not composed exclusively of PTMC patients. This sampling strategy is justified by the similarly favorable prognosis, outcomes, and quality of life across all low-risk, nonmetastatic thyroid cancer.^19–21^

Participants were recruited via email per our institutional review board policy, and verbal informed consent was obtained from each participant at the start of each study session. Participants received renumeration of $30 for their participation. All participants were enrolled between August 2022 and September 2024. All participants who initiated the study completed the full study protocol. This study was reviewed by our institutional review board and determined to be exempt.

### Analysis

Mean and median QALY weights across vignettes were compared using within-subjects repeated measures analysis of variance to assess differences in the uncomplicated vignettes, and paired Wilcoxon signed rank sum to assess differences between each pairing of uncomplicated versus complicated vignettes. Per published standards, the cohort was powered to detect a minimal important difference with an effect size of 0.5 (ie, 0.04 QALY).

Because the QALYs were not distributed normally for each health state, the data are not well represented by measures of central tendency and spread alone. Thus, we additionally estimated the parameters for beta distributions that approximate our QALY data, which is a common approach to represent the uncertainty around QALY values in cost-effectiveness analysis.^22^ The lower and upper bounds of the estimated distribution were set to 0 and 1 inclusive, respectively; any QALYs assigned to 0 or 1 were approximated as 0.0000000001 and 0.9999999999, respectively, to allow all QALY values to be within the beta distribution bounds for modeling purposes.

Following enrollment of the first 78 participants, a protocol change was implemented in which the shortest life span in perfect health shown to participants in the TTO exercise was adjusted from 0.5 (5 years) to 0 (0 years) to mitigate a theoretical floor effect of the instrument. The effect of this protocol change on QALY weights for each vignette was assessed by Wilcoxon rank-sum test. Mean QALY weights for each health state were similar before and after this protocol change (*P* = .05–.6103) for all scenarios except partial thyroidectomy, for which the median QALY was statistically (but not clinically) significantly higher for those using the instrument with a lower floor (0 years) (0.992 vs 0.988, *P* = .02).

We conducted 3 post hoc analyses. First, a subgroup analysis was conducted to evaluate the potential impact of participants’ own prior experience of treatment complications on their reported QALY weights for scenarios with complications. We computed a surgical complication composite QALY score by summing the absolute differences in QALY weights between complicated scenarios and their associated uncomplicated scenario. We then compared median composite scores between groups (participants who experienced complications vs patients who did not) using Wilcoxon rank sum. Second, we hypothesized that QALY assignment may vary based on the treatment experienced by the participants. To evaluate this question, we conducted a subgroup analysis comparing QALY weights for uncomplicated partial thyroidectomy between participants who underwent partial vs total thyroidectomy using Wilcoxon rank sum; the same was completed for uncomplicated total thyroidectomy QALY weights. Third, we conducted a subgroup analysis using paired Wilcoxon signed-rank tests to explore differences between uncomplicated QALY weights within subgroups of participants who (1) had personal history of PTMC and (2) had non-PTMC thyroid cancer.

*P* values of <.05 were considered to be statistically significant. SAS was used for all data analysis, including estimation of beta distribution parameters and visualization.

## Results

Data from 101 thyroid cancer survivors were collected; 24 (24%) were recruited through ResearchMatch.org. The median age of our sample was 54 years, and 73% were female. Clinical, demographic, and treatment characteristics of the participant cohort are described in Table I. Eighty-six percent of patients underwent total thyroidectomy and 30% experienced a treatment-related complication, suggesting that volunteers were enriched for those who had a more intensive treatment experience. Three (4%) of the cancer diagnoses were incidentally discovered on pathologic specimens for thyroid procedures conducted for another indication. Median length of time between first resection for thyroid cancer and participation in our study was 4 years (interquartile range 1.8–8.0) for all patients and 5 years (interquartile range 2.6–7.8) for those who experienced a treatment complication.

Median QALY weights for uncomplicated treatment scenarios ranged from 0.975 to 0.992 and were not significantly different between treatments (Table II; *P* = .15). Vignettes with treatment complications had significantly lower QALY weights across all treatment strategies (*P* < .01) except active surveillance complicated by progression requiring curative surgery (*P* = .72).

**Figure 1.**
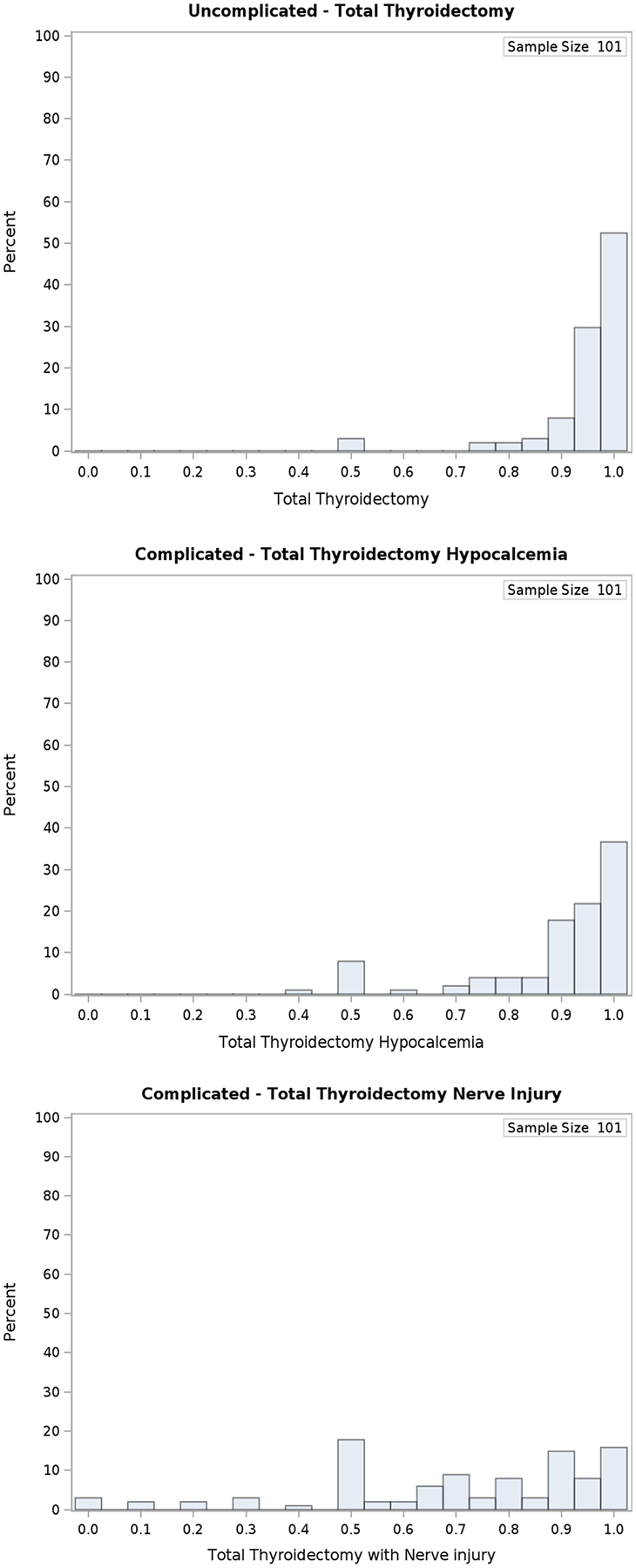
Distributions of quality-adjusted life year (QALY) weights for total thyroidectomy health states.

Representative beta distributions for the uncomplicated and complicated total thyroidectomy health states are shown in Figure 1. Beta distribution parameters for all health states’ QALY weights are included in Supplementary Table S1 and Supplementary Figure S1. These parameters can be incorporated into CEAs with the intent of probabilistic sensitivity analyses.

**Figure 2.**
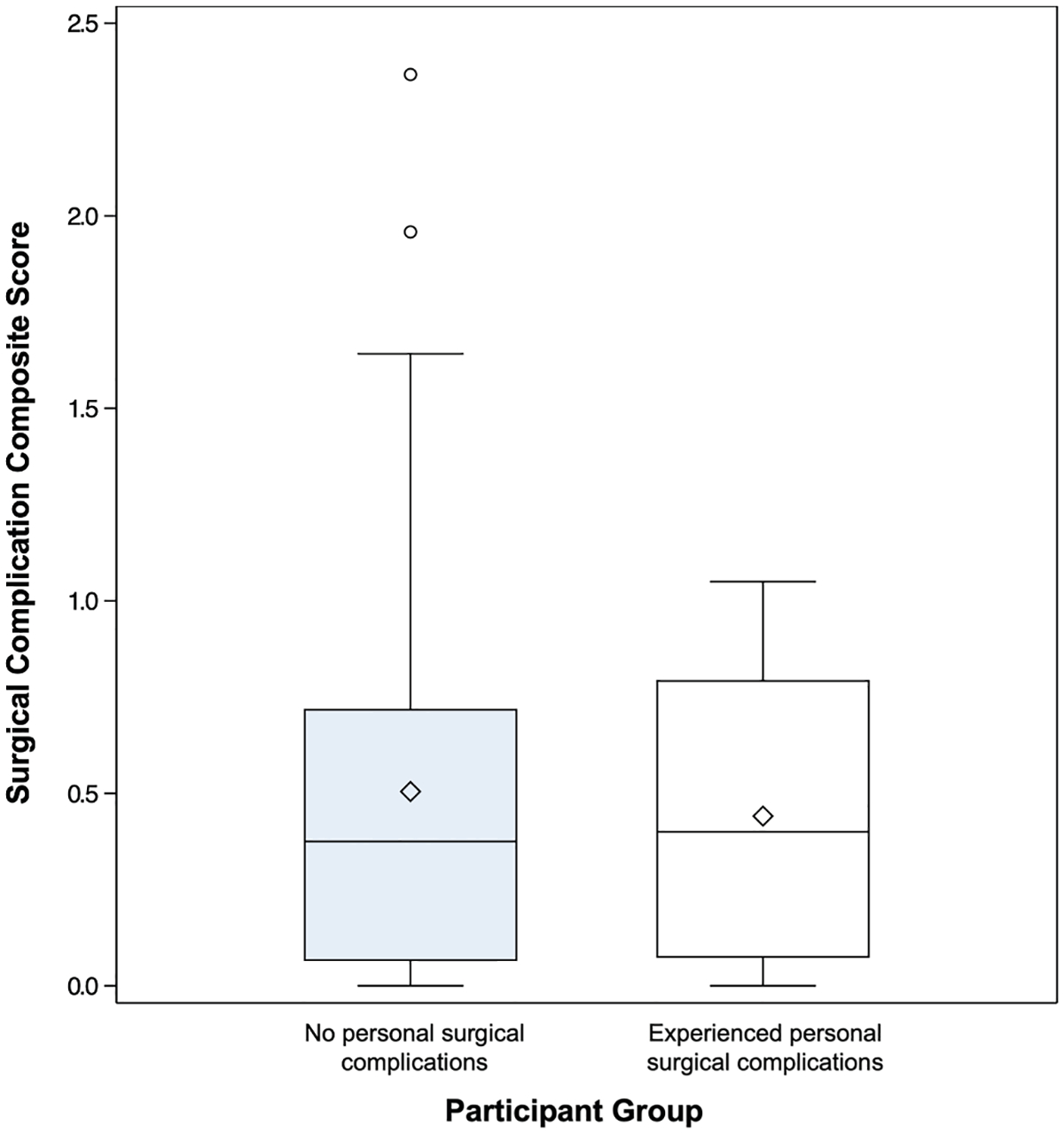
Surgical complication composite score, by participant personal experience of surgical complications.

The surgical complication composite score was similar between participants who had (*n* = 23) and had not (*n* = 54) themselves experienced complications (0.4 vs 0.375, respectively, *P* = .89), as shown in Figure 2. This suggests that personal experience of a treatment complication did not significantly impact the derivation of QALY weights.

QALY weights for surgical health states did not vary based on participants’ personal treatment history. The median QALY weights assigned to the uncomplicated partial thyroidectomy health state were similar between participants who underwent total (*n* = 66) vs partial thyroidectomy (*n* = 11) (medians: 0.992 vs 0.992, *P* = .94). This finding was echoed for the uncomplicated total thyroidectomy health state (0.967 vs 0.992, *P* = .88). Within the study cohort, 14 patients had a personal history of PTMC, whereas the remainder had non-PTMC disease. To determine whether participants’ personal cancer history influenced their preferences between different health states, we performed a post hoc subgroup analysis comparing health state QALY weights within these 2 subgroups. Overall, QALY weights for uncomplicated health states remained comparable to each other across both subgroups (Supplementary Table S2).

## Discussion

We derived QALY weights for 10 PTMC health states using TTO. Consistent with our hypothesis, QALY weights were similar across uncomplicated treatment states. Treatment-related complications were associated with lower QALY weight assignments for all treatments except active surveillance. This finding suggests that patient preferences in PTMC treatment are heavily driven by aversion toward complications, rather than the treatment pathways themselves. In the case of active surveillance, the fact that progression prompting resection did not result in a significant QALY decline is of particular interest. The implication is that participants did not view primary tumor growth on active surveillance to be a major distress, so long as curative resection remains achievable.

Our study reports the first collection of QALY weights derived using TTO administered to thyroid cancer survivors. Developing accurate cost-effectiveness models is critical in thyroid cancer, a disease with very high financial toxicity for patients. The out-of-pocket costs and bankruptcy rates associated with thyroid cancer are known to be among the highest among all cancer survivors.^23^ Several studies have documented the negative impact of financial strain on quality of life during cancer survivorship.^24,25^ For a disease in which treatment pathways are unlikely to affect long-term survival but could have divergent financial repercussions, treatment cost-effectiveness has real, patient-level implications. Deriving a robust set of QALYs is a necessary step toward identifying high-value treatments for thyroid cancer patients.

Overall, QALY weights derived using the time trade-off instrument in this study are higher than those previously reported in thyroid cancer. For example, QALY weights elicited via generic quality of life surveys from patients with current or history of differentiated or papillary thyroid cancer ranged from 0.711 to 0.994, but most were <0.95.^26^ There are several considerations that account for these differences. First, it has been reported that these generic assessments are not responsive in the thyroid cancer setting.^8^ Second, our study focuses exclusively on PTMC treatment scenarios. In another study, by Kebebew et al,^27^ QALY weights elicited from the general population for differentiated thyroid cancer scenarios using the standard gamble technique were lower than ours for unilateral nerve palsy (0.627 vs 0.95), hypoparathyroidism (0.778 vs 0.95), and bilateral nerve palsy (0.205 vs 0.75). These observed differences in QALY weights may be attributable to sampling differences (general population vs cancer survivors) and, as before, differences in the health states described (differentiated thyroid cancer vs specifically PTMC). Finally, Esnaola et al^28^ used TTO to elicit utilities from nurses and physicians for several low-risk PTC scenarios. Our surgical QALY weights are similar to this prior publication, which reported weights of 0.99 for lobectomy, 0.95 for total thyroidectomy, and 0.88 for any permanent complication. Our QALY weights are robust representations of patient preferences in PTMC because they were derived from a patient sample using gold standard methodology for health state descriptions specific to the very favorable long-term oncologic outlook of PTMC.

Uniquely, this study derived QALY weights from a sample of thyroid cancer survivors rather than from the general population. There is debate regarding the most appropriate sampling strategy for preference assessments.^29–31^ A general population sample may better capture the societal perspective of all taxpayers but may be prone to disability bias (overestimating burden of disease). Although a patient sample has the expertise of lived experience, it may downplay the impact of illness or may be willing to accept lower quality, higher cost interventions than the public would support. Undervaluation of health states by general population participants has been reported in the breast cancer literature,^32^ and our group is conducting a study to compare survivor-derived vs general population-derived QALY weights in thyroid cancer to further expand on these important considerations.

The optimal strategy for QALY weight derivation from patient samples is not well defined. For example, patient preferences may vary before, during, or after an index treatment. Our cross-sectional analysis would be well complemented by a prospective, longitudinal study that evaluates QALYs throughout the disease course in real-time. Similarly, it is unknown whether a respondent group must have personally experienced the precise health states in question in order to provide meaningful QALY weights. Indeed, it is rare for published QALY weights to be derived from such direct experience because of the specificity of individual health states. Here, we used a sample of early-stage thyroid cancer survivors to estimate the preferences of the PTMC population. Our post hoc analyses suggest that participants’ personal clinical history did not significantly impact preferences between treatments, suggesting that our results are a reasonable approximation of those that may be derived from a true PTMC population.

Our study has several limitations. First, potential confounders of QALY weight include age, socioeconomic status, and time since diagnosis.^8,33^ The impact of these factors requires further investigation with larger-scale, longitudinal studies. Second, our TTO methodology was modified over the course of the study (from a minimum value of 0.5 to 0) to avert a theoretical intrinsic floor effect. However, QALY weights were unchanged after this adjustment. Third, while considered a gold standard method for preference assessment, TTO has inherent limitations including a potential ceiling effect, comprehension, and interviewer effect.^34–36^ We mitigated the latter 2 limitations by including a tutorial and proctoring all interviews. Finally, most of our participants underwent total thyroidectomy for their cancer care. Although consensus guidelines generally advocate for more conservative approaches toward PTMC,^37^ it is notable that total thyroidectomy remains the most pervasive surgery performed for PTMC nationwide.^38^

In conclusion, this is the first report of patient-derived QALY weights for PTMC. QALY weights between treatment strategies were comparable, suggesting that patient preferences are heavily driven by aversion toward complications. As low-risk thyroid cancer management continues to de-escalate, understanding the cost-effectiveness of nonsurgical interventions will become increasingly critical. Our findings can contribute to resource allocation and shared decision making, particularly given the financial toxicity associated with thyroid cancer care.^23^ This article provides a set of robustly derived QALY weights for 10 low-risk thyroid cancer treatment scenarios to facilitate future cost-effectiveness research.

## Supplementary Material

supplementary material

Figure S1

1

Supplementary material associated with this article can be found, in the online version, at [https://doi.org/10.1016/j.surg.2025.109694].

## Figures and Tables

**Table I:** Participant characteristics (N = 101)

Characteristic	Value
Demographic characteristics	
Age, y	54 (42–66)
Sex: female	74 (73)
Race	
White or Caucasian	82 (81)
Black or African American	6 (6)
Asian	7 (7)
Other or prefer not to say	6 (6)
Ethnicity: non-Hispanic or Latino	97 (96)
Highest level of education completed	
High school	18 (18)
College	38 (38)
Postgraduate	44 (44)
Marital status: married	66 (65)
Employment status: employed	72 (72)
Household income: ≥$75,000	68 (70)
Insurance type: private insurance	70 (70)
Type of residence	
Urban	20 (20)
Suburban	65 (65)
Rural	15 (15)
Clinical characteristics (*n* = 77)	
Thyroid cancer size, cm	1.8 (1–3)
Preoperative clinical features*	
Extrathyroidal extension	2 (3)
Family history of thyroid cancer	6 (9)
Exposure to ionizing radiation	2 (3)
Compressive symptoms	24 (37)
Type of procedure	
Total thyroidectomy	66 (86)
Partial thyroidectomy	11 (14)
Pathology	
Papillary	69 (90)
Oncocytic	3 (4)
Follicular	3 (4)
Other	2
Lymph node positive	36 (47)
Years between treatment and survey completion	4 (2–8)
Complication	23 (30)
Postoperative thyroid replacement therapy	73 (95)

Continuous variables reported as median (q1–q3). Categorical variables reported as count (percentage). Clinical characteristics reported as *n* (%) of all patients with clinical data.

*IQR*, interquartile range.

* Not available for all patients; presented as % of nonmissing.

**Table II:** Quality-adjusted life year (QALY) weights for PTMC health states

Health state	QALY weight, median (IQR)	QALY weight, mean (SD)	*P*-value*	*P*-value^†^
Active surveillance	0.992 (0.933–0.992)	0.944 (0.092)		.15
Progression requiring surgery	0.983 (0.933–0.992)	0.945 (0.091)	.72	
Radiofrequency ablation	0.992 (0.950–1.000)	0.960 (0.081)		
Progression requiring surgery	0.983 (0.933–0.992)	0.945 (0.086)	<.01	
Temporary vocal cord palsy	0.983 (0.933–0.992)	0.944 (0.095)	<.01	
Partial thyroidectomy	0.992 (0.933–0.992)	0.948 (0.097)		
Permanent unilateral vocal cord palsy	0.950 (0.875–0.992)	0.895 (0.143)	<.01	
Total thyroidectomy	0.975 (0.942–0.992)	0.945 (0.094)		
Permanent hypocalcemia	0.950 (0.875–0.992)	0.887 (0.148)	<.01	
Permanent bilateral vocal cord palsy	0.750 (0.500–0.925)	0.706 (0.258)	<.01	

*IQR*, interquartile range; *QALY*, quality-adjusted life year; *PTMC*, papillary thyroid microcarcinoma; *SD*, standard deviation.

* Comparison between complicated and associated uncomplicated treatment.

† Overall comparison between uncomplicated treatments.
