# Supplementary material for: Patient preferences in papillary thyroid microcarcinoma management are driven by aversion toward complications rather than treatment pathway
Source: Surgery. Author manuscript; Available in PMC 2026 Jun 22. (PMC13222115; doi:10.1016/j.surg.2025.109694)
Supplement: supplementary material [file NIHMS2175625-supplement-supplementary_material.docx]

**Supplementary Material.**

**Table S1.** Beta Distribution Parameter Estimates for QALY Weights for PTMC Health States

|  | **Median** | **Mean** | **Standard Deviation** | **Alpha** | **Beta** |
| --- | --- | --- | --- | --- | --- |
| **Active Surveillance** | 0.997 | 0.948 | 0.112 | 2.766 | 0.150 |
| Progression requiring surgery | 0.995 | 0.948 | 0.104 | 3.394 | 0.187 |
| **Radiofrequency Ablation** | 0.999 | 0.962 | 0.091 | 3.332 | 0.132 |
| Progression requiring surgery | 0.996 | 0.949 | 0.106 | 3.157 | 0.170 |
| Temporary vocal cord palsy | 0.996 | 0.947 | 0.109 | 3.084 | 0.173 |
| **Partial Thyroidectomy** | 0.996 | 0.949 | 0.105 | 3.188 | 0.170 |
| Permanent unilateral vocal cord palsy | 0.980 | 0.903 | 0.160 | 2.199 | 0.237 |
| **Total Thyroidectomy** | 0.994 | 0.947 | 0.104 | 3.490 | 0.196 |
| Permanent hypocalcemia | 0.969 | 0.894 | 0.160 | 2.407 | 0.286 |
| Permanent bilateral vocal cord palsy | 0.743 | 0.619 | 0.367 | 0.465 | 0.287 |

*Footnote****:*** QALY = Quality Adjusted Life Year. PTMC = Papillary Thyroid Microcarcinoma. Means, medians, and standard deviations reported here are those of the beta distribution model, not of the study sample.

**Table S2.** QALY Weights for Uncomplicated PTMC Health States, by PTMC Status

|  | **n** | **Active Surveillance** | **RFA** | **Lobectomy** | **Total Thyroidectomy** | **p-value** |
| --- | --- | --- | --- | --- | --- | --- |
| **PTMC** | 14 | 0.925  (0.80-0.992) | 0.979  (0.908-0.992) | 0.933  (0.808-0.983) | 0.942  (0.842-0.983) | 0.03-0.92 |
| **Non-PTMC** | 63 | 0.992  (0.933-1.0) | 0.992  (0.950-1.00) | 0.992  (0.942-0.992) | 0.967  (0.950-0.992) | 0.07-0.91 |

*Footnote****:*** QALY = Quality Adjusted Life Year. PTMC = Papillary Thyroid Microcarcinoma. PTMC was defined as tumor <1cm and no additional risk factors (e.g. extrathyroidal extension, family history of thyroid cancer, prior exposure to ionizing radiation). The PTMC participants theoretically would have been eligible for all 4 treatment pathways, whereas the non-PTMC participants would theoretically have been eligible for partial and/or total thyroidectomy only.
