# Supplementary figures and images for: Patient preferences in papillary thyroid microcarcinoma management are driven by aversion toward complications rather than treatment pathway
Source: Surgery. Author manuscript; Available in PMC 2026 Jun 22. (PMC13222115; doi:10.1016/j.surg.2025.109694)

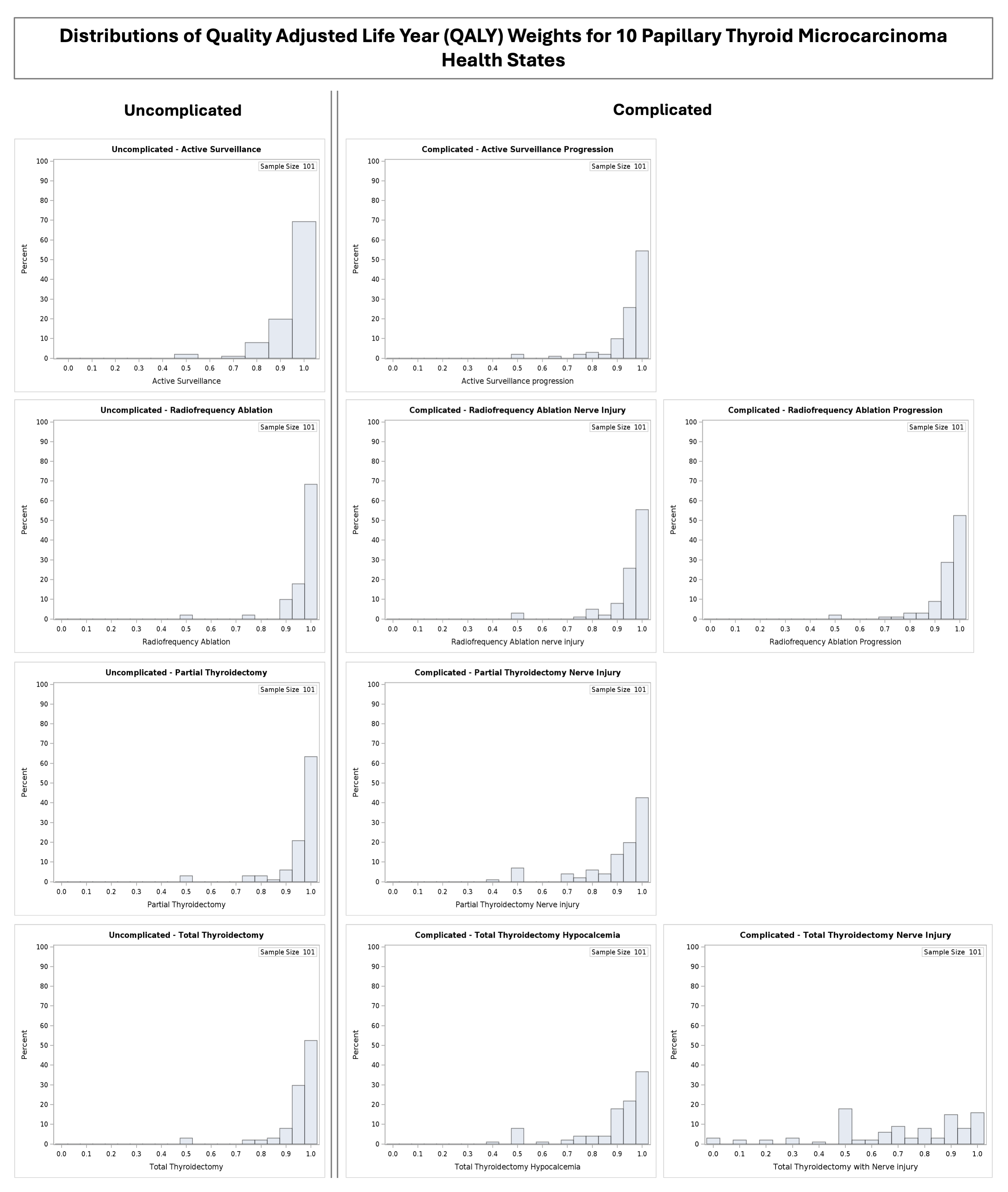

Supplement: Figure S1 [file NIHMS2175625-supplement-Figure_S1.tiff]
