# Supplementary material for: Patient preferences in papillary thyroid microcarcinoma management are driven by aversion toward complications rather than treatment pathway
Source: Surgery. Author manuscript; Available in PMC 2026 Jun 22. (PMC13222115; doi:10.1016/j.surg.2025.109694)
Supplement: 1 [file NIHMS2175625-supplement-1.pdf]

Discussion

Dr Quan Duh (University of California, San Francisco): Have you thought about doing the same study for people that have undergone radiofrequency ablation for PTMC, and/or have undergone active surveillance. My concern with a study like this is that you have a selected population that have already undergone an operation. So, can you comment on that?

Rebecca Kowalski: Certainly, this is the beginning of our study and as nonoperative cases for low-risk thyroid cancer expand, we could accrue additional participants to study this. The evidence here would suggest that the QALY weights are not affected by the extent of operative intervention, comparing partial versus total thyroidectomy subgroups. Exploring this in a nonoperative group would be a logical next step.

Susan Pitt, MD (University of Michigan, Ann Arbor): Disclosure: I received some funding from Johnson and Johnson for consulting work unrelated to this.

I think this is a wonderful talk, and thank you so much for putting really good, great methodology into patient preferences. How confident are you that patient preferences are driven by complications? If you interview patients prior to their treatment

and prior to their decision on whether or not to undergo surgery, they will usually think about things like survival or recurrence, and minimize the import of complications.

Rebecca Kowalski: We are confident that our data appropriately represent the evaluated health states. However, the findings may not be generalizable beyond these health states, for example, in larger or higher-risk cancers, for which survival or recurrence is a greater concern. Another way to explore this might be repeating this study with participants prior to any kind of treatment to see if preferences change throughout the process. Regardless, the data are clear that preferences were consistently different between health states that were uncomplicated and complicated.

Dr Insoo Suh (New York University Langone Health, New York): Congratulations on an excellent study. I noticed in your RFA arm, the maximal disutility state was a temporary vocal cord palsy. I'm wondering what led you to use that as opposed to a permanent palsy? I'm sure that in expert hands, the most likely poor-outcome scenario you're going to have is a temporary vocal cord palsy. But as this technique is adopted further into less and less experienced hands, I can imagine that the average practitioner may be more likely to run into misadventures.

Rebecca Kowalski: We chose temporary nerve palsy because it is the more commonly encountered scenario with RFA, from the data we've seen so far. Future research in this area could include more uncommon but permanent outcomes as RFA becomes more widely used.

Chris O'Neill, MD (University of Newcastle, Newcastle): My question follows on from Dr Pitts. We know in preference studies that fear of cancer recurrence is a very important issue in both decision making and then subsequently in quality-of-life outcomes. It's great to see you develop QALYs for complications. But how do we put fear of cancer recurrence into that framework?

Rebecca Kowalski: The cancer progression health state for RFA and active surveillance treatments may get at a similar fear of recurrence, but ultimately, you're right, we do not cover recurrence among our health states. That's a great thought for us as we evaluate additional health states in the future.

Kyle Zanocco, MD (University of California, Los Angelos): Did you include patients that actually had a complication, like a recurrent nerve injury? And if you did, some economists argue that when you ask patients that have the condition, they will kind of upregulate or say it wasn't as bad as the general population thinks it is. And a lot of times they suggest that you exclude patients that actually have the complication. So can you comment on that?

Rebecca Kowalski: Thank you for the question. Actually, I may have some data that might help. We did include patients with complications, and the complication rate across our participants was approximately 30% among those for whom we had clinical data. We made a composite score of the discrepancies between QALY weights for each complicated and uncomplicated health state pair. Then, we compared that score between those who had experienced a complication and those who did not. We found that there was really no difference. Although not conclusive, it's at least an exploration into that space, and definitely something that we can explore with a larger sample size in the future.
